# **Microbial circadian clocks: host-microbe interplay in diel cycles**

**DOI:** 10.1186/s12866-023-02839-4

**Published:** 2023-05-09

**Authors:** Emily M. Wollmuth, Esther R. Angert

**Affiliations:** Department of Microbiology, Cornell University, 123 Wing Drive, Ithaca, NY 14853 USA

**Keywords:** Circadian rhythm, Circadian clock, Diurnal cycle, Diel cycle, Oscillations, Symbiosis, Gut microbiota

## Abstract

**Background:**

Circadian rhythms, observed across all domains of life, enable organisms to anticipate and prepare for diel changes in environmental conditions. In bacteria, a circadian clock mechanism has only been characterized in cyanobacteria to date. These clocks regulate cyclical patterns of gene expression and metabolism which contribute to the success of cyanobacteria in their natural environments. The potential impact of self-generated circadian rhythms in other bacterial and microbial populations has motivated extensive research to identify novel circadian clocks.

**Main text:**

Daily oscillations in microbial community composition and function have been observed in ocean ecosystems and in symbioses. These oscillations are influenced by abiotic factors such as light and the availability of nutrients. In the ocean ecosystems and in some marine symbioses, oscillations are largely controlled by light-dark cycles. In gut systems, the influx of nutrients after host feeding drastically alters the composition and function of the gut microbiota. Conversely, the gut microbiota can influence the host circadian rhythm by a variety of mechanisms including through interacting with the host immune system. The intricate and complex relationship between the microbiota and their host makes it challenging to disentangle host behaviors from bacterial circadian rhythms and clock mechanisms that might govern the daily oscillations observed in these microbial populations.

**Conclusions:**

While the ability to anticipate the cyclical behaviors of their host would likely be enhanced by a self-sustained circadian rhythm, more evidence and further studies are needed to confirm whether host-associated heterotrophic bacteria possess such systems. In addition, the mechanisms by which heterotrophic bacteria might respond to diel cycles in environmental conditions has yet to be uncovered.

## Background

Circadian rhythms are observed across all domains of life and can enable organisms to anticipate and prepare for daily changes in their environment [1]. While many biological processes occur on an approximately 24-hour cycle, circadian rhythms are distinct from diel cycles in that they are self-sustained and free-running. Although circadian rhythms are entrained to environmental stimuli, such as light-dark cycles, these endogenous rhythms are temperature compensated—that is, capable of maintaining rhythmicity on a nearly 24-hour cycle despite shifts in temperature. Circadian rhythms were long believed to be exclusively controlled by complex transcriptional-translational feedback loops involving intertwined positive and negative regulatory branches. The positive branch is made up of activator proteins which promote transcription of one or more repressor proteins of the negative branch. These negative-branch repressor proteins then inhibit further transcription of positive-branch activators, resulting in recurrent oscillations in gene expression that drive the circadian rhythm [2, 3]. Circadian clocks have been characterized in many microbial eukaryotes, including the fungi *Saccharomyces cerevisiae* and *Neurospora crassa*, and the importance of these clock mechanisms in regulating energy metabolism, cell growth, and physiology is well documented [3–5].

While transcriptional-translational feedback loops play important roles in circadian rhythms, several studies have uncovered alternative feedback systems. It has been demonstrated that human red blood cells can sustain a circadian rhythm in the absence of gene expression, suggesting that cellular circadian clocks might be controlled by redox reactions related to metabolism [6, 7]. Circadian rhythms can also persist in knockout mice where key feedback loop transcription factors are absent [8–10]. Knowledge about the role of posttranscriptional regulation of circadian rhythms—such as those mediated by non-coding RNAs—has expanded in recent years, further supporting the idea that circadian rhythms are controlled by a diverse array of molecular mechanisms [11–13].

The antioxidant enzyme peroxiredoxin regulates peroxide levels within eukaryotic cells and maintains a self-sustained oscillation in its redox state in the absence of any transcriptional-translational feedback [8]. The phenomenon of peroxiredoxin redox cycling has been observed in diverse model organisms, including *Arabidopsis thaliana*, *Neurospora crassa, Ostreococcus tauri*, and *Synechococcus elongatus* [14]. Putative peroxiredoxins are observed in over 50 bacterial phyla [15] suggesting one potential mechanism by which diverse bacteria might detect environmental fluctuations to influence and maintain diel oscillations. However, in bacteria, a circadian clock mechanism has only been characterized in cyanobacteria [16]. Remarkably, a functioning version of the cyanobacterial clock has been reconstructed in vitro, in the absence of gene expression [17]. The discovery of these simple protein-based circadian clocks raises the possibility that other bacteria possess internal circadian clocks that have yet to be described. The ability to anticipate daily environmental changes could convey an evolutionary advantage to single-celled organisms. In fact, diverse host-associated microbes—including the skin microbiota of rainbow trout, root-associated microbes of the rhizosphere, the human oral microbiota, and malarial parasites—have been suggested to modulate host circadian rhythms or to possess their own diel rhythms [18–21]. In particular, microbes residing in gut environments may benefit from being able to anticipate and prepare for the daily behaviors of their host and influxes of nutrients [22].

Many studies have examined the relationship between gut bacterial communities and host circadian rhythms and have provided insights into the oscillations in microbiota composition or metabolic activity that occur on a 24-hour basis [23, 24]. However, studying the rhythmicity of gut systems is technically challenging. Considering the impacts of diverse abiotic factors on gut systems, sufficient sampling frequency, and data collection through multiple consecutive cycles are imperative to accurately characterize biological rhythms [25]. Additionally, the complexity of gut systems raises a question that currently lacks a clear answer: do endogenous circadian cycles of gut bacteria modulate host circadian rhythms or are microbial cycles governed only by the circadian rhythms of their hosts and influential abiotic factors?

This review will describe what is known about diel cycles and circadian rhythms in bacteria, covering the well-characterized Kai system in cyanobacteria, microbial oscillations observed in aquatic systems, and the influence of abiotic factors such as light and host feeding behaviors in gut environments. Current evidence of the impact of host circadian rhythms and behaviors on bacterial diel cycles will also be presented. Finally, it will outline recent discoveries about the influence cyclical patterns in the microbial gut community may have on host rhythms. Although host-associated eukaryotic microbes and archaea likely influence host circadian rhythms, most gut microbiome work to date has focused on bacteria. Thus, this review will primarily discuss bacterial rhythms while including some relevant examples involving eukaryotic microbes or archaea. For additional depth on many of these topics, see the book *Circadian Rhythms in Bacteria and Microbiomes* [26].

## Circadian clocks in cyanobacteria

Circadian rhythms were long considered to be exclusive to eukaryotes as bacteria were believed to be too simple to sustain a complex clock system [16]. This view was challenged when a circadian clock controlling nitrogen fixation and the uptake of amino acids was observed in the cyanobacterium *Synechococcus* sp. RF-1 [27–29]. The circadian rhythms of cyanobacteria have since been further elucidated. Genetic work focusing on the model organism *S. elongatus* unveiled a cyanobacterial clock controlled by a negative feedback system involving three proteins, KaiA, KaiB, and KaiC, which are responsible for genome-wide oscillations in gene expression [30].

**Fig. 1:**
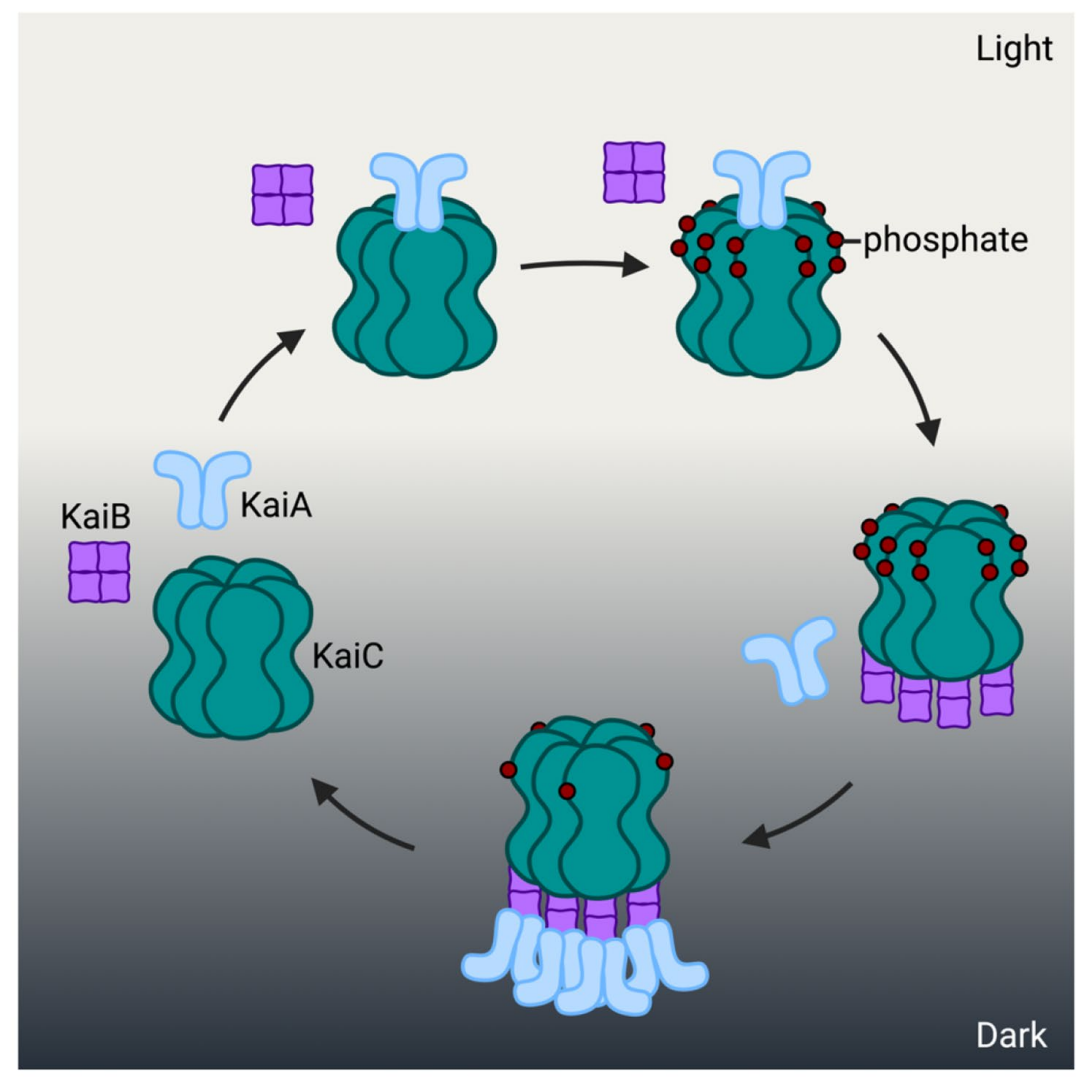
Kai protein interactions in *S. elongatus* The core clock components, KaiC peptides, assemble into a ring-shaped hexamer that acts as an ATPase as each subunit undergoes cycles of phosphorylation and dephosphorylation. During the day, KaiA subunits repeatedly bind to the KaiC hexamer promoting autophosphorylation of KaiC. In the evening, KaiB peptides bind the hyperphosphorylated KaiC hexamer. The bound KaiB peptides simultaneously sequester KaiA and prevent it from interacting with KaiC in a manner that would promote autophosphorylation of KaiC overnight. When KaiC is sufficiently dephosphorylated, it releases the KaiAB complex, which allows the cycle to begin again. In the figure, the day-night cycle is indicated by a change in background; dark gray indicates night. The phosphorylation state of KaiC is indicated by the number of bound phosphates shown as small circles. Created with BioRender.com.

Biochemical and protein structural analyses have provided details about the basis for this endogenous circadian system (Fig. 1). KaiC peptides spontaneously assemble into a ring-shaped hexamer [31, 32]. During the daytime, KaiA binds KaiC to promote its autophosphorylation [33, 34]. In the evening, KaiB binds to KaiC while simultaneously sequestering KaiA, preventing further interactions with KaiC. With the direct association between KaiA and KaiC blocked, the phosphorylation state of the KaiC hexamer deteriorates overnight [34–36]. Full dephosphorylation leads to the release of KaiB which in turn frees KaiA [34]. KaiC and KaiA are free to interact and repeat the cycle. Perhaps most striking is the ability to reconstruct a functional cyanobacterial clock outside the cellular environment. The cyclical interactions of KaiA, KaiB, and KaiC have been reproduced in vitro with recombinant proteins mixed together in the presence of magnesium and ATP [17]. Simply the addition of ATP is enough to initiate the 24-hour KaiC phosphorylation-dephosphorylation cycle [17].

In *S. elongatus* where KaiA is inactivated, circadian oscillations are severely dampened, indicating the importance of KaiA for maintenance of an endogenous rhythm [37]. In regions of the globe where day length changes seasonally, conditions remain cyclical, but the entrainment signal provided by light is less reliable, thus more robust circadian clock systems may be needed for consistent input to sustain metabolic stability. The full Kai system of *S. elongatus* is capable of tracking the 24-hour day-night cycle regardless of the length of day [38]. However, variations of the Kai system lacking KaiA have been observed in other cyanobacteria and purple bacteria, and these modified systems appear molded for particular lifestyles [39, 40]. In *Prochlorococcus marinus*, a small cyanobacterium typically found in tropical and subtropical open ocean environments, a Kai system which lacks KaiA has been identified. This simplified system has been described as a timer rather than a clock. While this system still responds to environmental signals, unlike an endogenous circadian rhythm, cycles in KaiC phosphorylation have been experimentally shown to cease immediately in the absence of lightdark cycle entrainment [40]. The existence of this simplified Kai system is likely a consequence of selection for genome reduction driven by adaptation of *Prochlorococcus* to low-nutrient ecosystems [41]. It has also been hypothesized that a strong, self-sustaining circadian clock may be unnecessary near the equator where environmental cycles are generally more stable year-round [40, 42]. This could apply to timekeeping mechanisms used in other stable environments as well. Although such variations of the Kai system do not function as true circadian clocks in that they cannot maintain endogenous rhythms, these systems may still be important for bacteria in responding to cyclical environment cues in a variety of habitats. Other cyanobacteria also possess variations of the standard KaiABC clock mechanism. *Synechocystis* sp. PCC 6803, for example, uses a Kai system with multiple variants of KaiB and KaiC. This system has been suggested to function as a timer that runs in addition to the primary KaiABC clock [43]. Understanding these nuances and their impact on selection for the configuration of a circadian clock may provide guidance for seeking novel timekeeping mechanisms in bacteria more broadly [40].

The Kai system temporally separates the expression of incompatible functions—oxygenic photosynthesis during the day and the oxygen intolerant fixation of nitrogen at night—and thus conserves energy and resources by not producing the nitrogenase enzyme under conditions that would require it to be recycled. The Kai system also controls a variety of cellular functions including timing of cell division, energy management, and natural competence [44–47]. Consequently, circadian clock systems allow organisms to optimize the timing of physiological processes [1]. Considering the potential advantages such systems might provide, it is not surprising that there is evidence that suggests similar timekeeping mechanisms may exist in other microbes. Homologs of *kai* genes have been identified in other cyanobacteria as well as in Archaea, Pseudomonadota (synonym Proteobacteria), and Chloroflexi [48, 49]. Although *kai* homologs may be indicative of circadian function, their presence does not necessarily show that these taxa possess a circadian clock. There is evidence that Kai-like proteins may also be involved in other cellular processes such as stress response and biofilm formation [50], perhaps explaining their widespread presence in the genomes of diverse bacterial taxa.

The potential value of Kai-based regulatory systems has generated a growing body of experimental work designed to test the performance of Kai systems. For example, a fully functional Kai system has been successfully expressed in *Escherichia coli* [51]. In the model cyanobacterial system, exposure to darkness during the day results in a circadian phase shift that is paired with a significant reduction in the proportion of ATP in the adenine nucleotide pool, suggesting energy metabolism entrains the circadian rhythm of *S. elongatus* [52]. In a genetically rewired *S. elongatus* that can use either photosynthesis or glucose supplied in the environment, rhythmic feeding of glucose was sufficient to drive metabolic cycles that in turn entrained the Kai clock in the absence of light [53].

In addition, metatranscriptomic studies have provided some evidence of diel expression cycles in non-photosynthetic members of the Pseudomonadota and Bacteroidota (synonym Bacteroidetes), residing in microbial mats alongside Cyanobacteria [54]. The authors suggest that *kai* genes recovered from non-phototrophic members of the mats may indicate the presence of circadian clocks that regulate fluctuating expression of some of their genes. However, light, metabolic inputs from neighboring cells, and daily temperature fluctuations were not explored as potential regulatory signals. Instead, the authors describe these only as potential Kai system entrainment signals [54]. These heterotrophic bacteria may be displaying a circadian rhythm, or alternatively they may simply be responding to the diel influx of fixed carbon and oxygen resulting from the photosynthesis of their cyanobacterial associates.

Diel oscillations have also been observed in heterotrophic bacteria growing in monoculture in the lab environment, even in bacteria with no known *kai* homologs. In *Bacillus subtilis*, an approximately 24-hour cycle in promoter activity for *ytvA*, a gene that encodes for a blue light photoreceptor, can be entrained to light-dark cycles, and expression continues to cycle when the temperature is modified within a 6 °C range [55]. Similarly, *Klebsiella aerogenes* displays oscillations in the expression of the motor protein gene *motA* on a roughly daily cycle, which results in pulses of swarming motility [56, 57]. These findings, in combination with the existence of simple Kai timers, demand further inquiry of the drivers of diel metabolic oscillations in aquatic microbial communities and nonphotosynthetic bacteria [40].

## Diel oscillations in aquatic microbes

Cyclical oscillations in gene expression and metabolism are prevalent among bacteria and free-living, single-celled eukaryotes in open-ocean microbial communities [58, 59]. These daily oscillations are impacted by light intensity and quality [60], and are well documented in marine phototrophs such as cyanobacteria, purple bacteria, and picoeukaryotic alga [61–64]. However, perhaps more intriguingly, diel transcriptional changes occur in heterotrophs as well [58, 65].

Many aquatic bacteria—including some heterotrophs—are capable of light sensing via photosensory proteins [66]. Photoheterotrophic bacterioplankton, including *Roseobacter* and *Pelagibacter*, display diel cycling in their gene transcription patterns which resemble oscillations observed of aquatic photoautotrophs [59]. Daily oscillations in photoheterotrophs could be regulated in part by light detected by proteorhodopsin photosystems. Rhodopsins consist of an opsin protein covalently linked to the chromophore retinal. Proteorhodopsins found in bacteria absorb photons used to produce energy or initiate intracellular signaling [67, 68]. These light-driven ion pumps are often employed by photoheterotrophs to supplement the energy they derive from organic carbon [69, 70]. Proteorhodopsins have also been shown to help microorganisms cope with suboptimal environmental conditions such as limited nutrient availability [69, 71, 72]. The diverse array of proteorhodopsin variants observed in the ocean contribute significantly to the total solar energy captured by phototrophs in surface waters globally [73, 74]. Rhodopsins have also been observed in saline lakes and freshwater ecosystems [75–77]. One study of freshwater Actinomycetota (synonym Actinobacteria) found that light altered the expression of transport proteins and enhanced the growth rate of the bacteria, suggestive of a cyclical light-induced response [78]. However, the proteorhodopsin photosystems of these bacteria were not responsible for this increased growth, indicating other photosystems may also be important for modulating daily metabolic responses [78].

Viruses serve as vectors for distributing genes among oceanic microbes [79]. Genes for rhodopsin photosystems have been observed in the genomes of several giant viruses, and one study suggested that a choanoflagellate collected from the wild gained its phototrophic capabilities either from infection with or by feeding on such a virus [80, 81]. The diel cycles of diverse phototropic and heterotrophic microplankton are influenced by viral infection, with both viral and host transcription peaking during the day [65]. In bacterioplankton, phages—viruses that infect bacteria—have been shown to alter cyanobacterial metabolism by supplementing photosynthetic machinery during infection [82, 83]. Similar effects are also seen in non-phototrophs, where phages have been shown to alter community diversity and function by introducing genes encoding new metabolic traits [79]. Replication cycles of prevalent oceanic viruses have also been shown to synchronize with the diel metabolic and reproductive cycles of their microbial hosts [84–86].

Light sensing and viral mediation of metabolism are both significant drivers of diel oscillations in oceanic microbial communities. These capabilities have been shown to influence microbes in other systems as well, including in laboratory culture. Light impacts stress responses, motility, and biofilm formation of many non-phototrophic bacteria [87–89] and bacteriophages have been shown to alter bacterial virulence and biofilm formation [90, 91]. Such findings highlight that metabolically and phylogenetically diverse bacteria may respond to environmental stimuli similarly. Microbes residing in diverse environments are exposed to light-dark cycles and cyclical pulses of nutrients, thus both free-living and host-associated microbes must be able to respond to these changes to be competitive. These nearly universal responses to environmental fluctuations emphasize the need for further study of the impacts of photosystems, stress responses, metabolism, and viruses on microbial diel cycles in a wide range of environments including in host-associated microbial communities.

## Interplay between host and symbiont diel cycles

Symbiotic microbes influence the diel cycles of their host across a diverse array of systems including in both plant and animal hosts. In plants, the rhizosphere serves as an interface between the roots and nearby soil microbes that may facilitate access to nutrients, such as usable nitrogen, phosphorus, and a variety of minerals [92]. There is growing evidence that the circadian rhythm of the host plant influences microbial activity in the rhizosphere which likewise feeds back to the plant in a bidirectional relationship. In the model organism *A. thaliana*, mutations in key circadian clock genes have been shown to alter oscillations in the abundance of both fungal and bacterial species in the rhizosphere community [93, 94]. Similarly, in rice (*Oryza sativa* L.) disruption of the host circadian rhythm by altering the light-dark cycle, led to changes in which rhizosphere-associated bacterial species display diel oscillations in abundance [95]. Recent work in *A. thaliana* and its wild relative *Boechera stricta* demonstrated that the rhizosphere microbes also influence host circadian rhythms. In plants where the rhizosphere microbial community was disrupted, the length of the circadian period was extended, lasting longer than 24 h, suggesting a role for the microbial community in regulating host circadian rhythms [20].

**Fig. 2:**
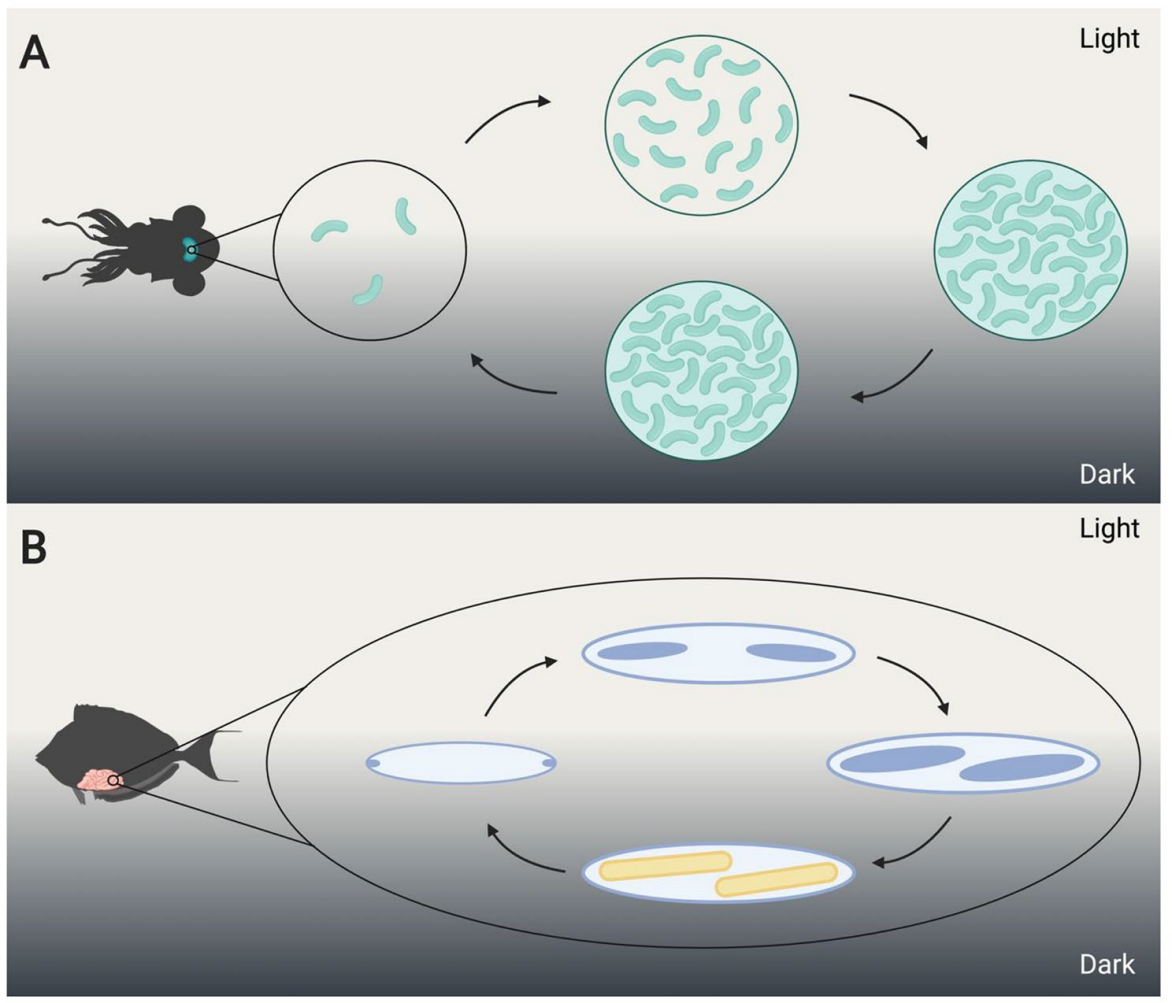
Host and symbiont diel cycles in diverse marine organisms (A) Each day at dawn, the majority of the resident *Allivibrio fischeri* population is expelled from the *Euprymna scolopes* light organ resulting in low symbiont population density (illustrated by the number of bacteria in the circle). Throughout the day, the remaining symbiont population grows to achieve the necessary density for light production by evening. (B) *Epulopiscium* sp. type C produce multiple intracellular offspring on a diel cycle. Each morning, the cell divides at each pole. Over the course of the day, the intracellular offspring (forespores) grow. Mature endospores are released from the mother cell prior to sunrise and the spores germinate. The day-night cycle is indicated by a change in background; dark gray indicates night. Forespores are represented by filled in blue ovals and endospores are filled in yellow. Created with BioRender.com.

Synchrony between host and symbiont metabolic cycles has been described in several marine systems as well. The association between the Hawaiian bobtail squid *Euprymna scolopes* and the bacterium *Allivibrio fischeri* provides a valuable model used to study topics ranging from host-symbiont coevolution to the genetic mechanisms responsible for establishing a mutualism in every generation [96]. The daily activity of the host squid dictates the metabolism and population dynamics of *A. fischeri*. The bioluminescent *A. fischeri* are housed in the light organ of the squid host [97]. The light emitted from the light organ is modulated by the host and is used for counter-illumination camouflage to evade predators of the squid when it is active at night [98]. Each dawn, most of the bacterial population is expelled from the light organ, and the host hides, buried in the sand. The small population of symbionts remaining in the light organ must grow rapidly to achieve the necessary density for light production by evening (Fig. 2A), in time for the nocturnal host to become active again [97].

In a study of captive squid, it was observed that the hemolymph metabolome varied temporally, likely influenced by the changing abundance of symbionts in the light organ [99]. In the same study, squid deprived of their symbiont displayed altered abundance of metabolites in the hemolymph compared to symbiont-possessing squid [99]. In wild-caught squid, gene expression studies have uncovered transcriptional shifts that accompany the daily changes in symbiont populations [100, 101]. For the host, immune-related and stress response genes along with genes for cytoskeletal functions increase in expression in the light organ just before dawn. These changes in expression presage changes that occur in the light organ as the epithelial lining deteriorates just before the expulsion of symbionts [100]. The symbionts remaining in the light organ adjust their metabolism to glycerol use [101]. Host cytoskeletal gene expression drops within a few hours after dawn [100]. As the lining of the light organ recovers, symbiont populations increase. By dusk, gene expression in the symbiont population changes to repress glycerol use while expression of genes associated with catabolism of chitin increases [100, 101]. Together the host and symbiont control the migration of host hemocytes, which provide chitin to fuel symbiont population growth. The squid regulates production of macrophage migration inhibitory factor (MIF). During the day, a high MIF level inhibits hemocytes from entering the light organ. At night, chitin breakdown and fermentation by the symbiont produces catabolites such as acetate, lactate, pyruvate, and succinate which attract hemocytes to the light organ [102]. Further, *A. fischeri* may directly influence the circadian clock of *E. scolopes*. The expression of a host circadian rhythm related gene, *cry1*, may be influenced by the light emitted by the bioluminescent symbiont community [103]. Cry1 is a repressor protein in the negative branch of the circadian feedback loop that is upregulated in the squid light organ at night, aligning with the increase in bacterial bioluminescence [103]. Strikingly, both symbiont-deficient squid and squid that possess genetically modified non-bioluminescent symbionts do not show the same diel oscillation in expression of *cry1* observed in squid that possess the wild-type bioluminescent symbionts [103]. Together, these findings suggest that the diel cycles of *E. scolopes* and *A. fischeri* are intertwined, modulating one another.

The diel metabolic cycles of giant clams and their photosynthetic symbiotic partners also appear to occur contemporaneously. The symbiont *Symbiodinium* sp. provides energy for its host *Tridacna* sp. via release of carbohydrates such as glucose and glycerol that the host can metabolize [104]. The concentration of glucose in the clam hemolymph cycles on a diel basis, with maximum daytime levels 3.2-fold higher than the overnight low, aligning with the photosynthetic production of glucose by the symbiont [105]. Experimental changes in light-dark cycles have been associated with a reduction in photosynthetic performance by the symbiont and changes in the expression of clock genes in the host [106]. A molecular study showed that a glycerol production gene expressed in both symbiotic and free-living *Symbiodinium* is not expressed overnight [107], alluding to the significance of *Symbiodinium* for controlling the daily metabolic cycle of the host. *Symbiodinium* is also an important nutritional symbiont for some corals such as *Euphyllia paradivisa*. One recent study that examined the temporal pattern of gene expression in *E. paradivisa* found the host circadian clock continued to function in the absence of the symbiont. However, various genes displayed an altered circadian period in the absence of the symbiont, suggesting that *Symbiodinium* modifies the host circadian rhythm [108].

Coordination between host and symbiont diel rhythms is also observed in surgeonfish (Family Acanthuridae) and their gut associated symbionts *Epulopiscium* spp. and related bacteria called “epulos”. This group of bacteria, within the Lachnospiraceae family, are often prevalent members of the gut microbial communities of surgeonfish and are important for host nutrition by mediating the digestion of plant material through carbohydrate breakdown and fermentation reactions [109]. *Epulopiscium* sp. type C (Fig. 2B) and type J display a diel reproductive cycle which corresponds with host rhythms. The cycle culminates in the nocturnal production of endospores which protects the bacteria from nutrient deprivation while the host fasts overnight [110, 111]. Early work suggested that *Epulopiscium* sp. type A reproduction, motility, and growth follow a diel cycle [111]. These observations have since been supported by differential expression studies, where diel oscillations in transcription of genes related to carbon and nitrogen metabolism, sporulation, and motility were observed (FA Arroyo, personal communications).

As these symbioses demonstrate, the entangled diel oscillations of host and symbiont are most often heavily influenced by light-dark cycles that impact host behavior and nutrition. Alterations in light-dark cycles impact the circadian rhythm of plants, which changes the microbial community composition of the rhisophere. In the case of the bobtail squid, the host cultivates its symbiotic bacteria to optimally produce light which may in turn alter host circadian rhythms. The daily metabolic cycles observed in giant clams are dependent on the photosynthetic activity of their symbionts, and epulos in the surgeonfish gut grow and sporulate in synchrony with host feeding and fasting. The significance of host diet and feeding as well as light-dark cycles in influencing these associations holds true in other systems as well, playing an important role in the interaction between animals and their gut microbiota.

## Light-dark cycles impact gut microbial community composition and host physiology

**Fig. 3:**
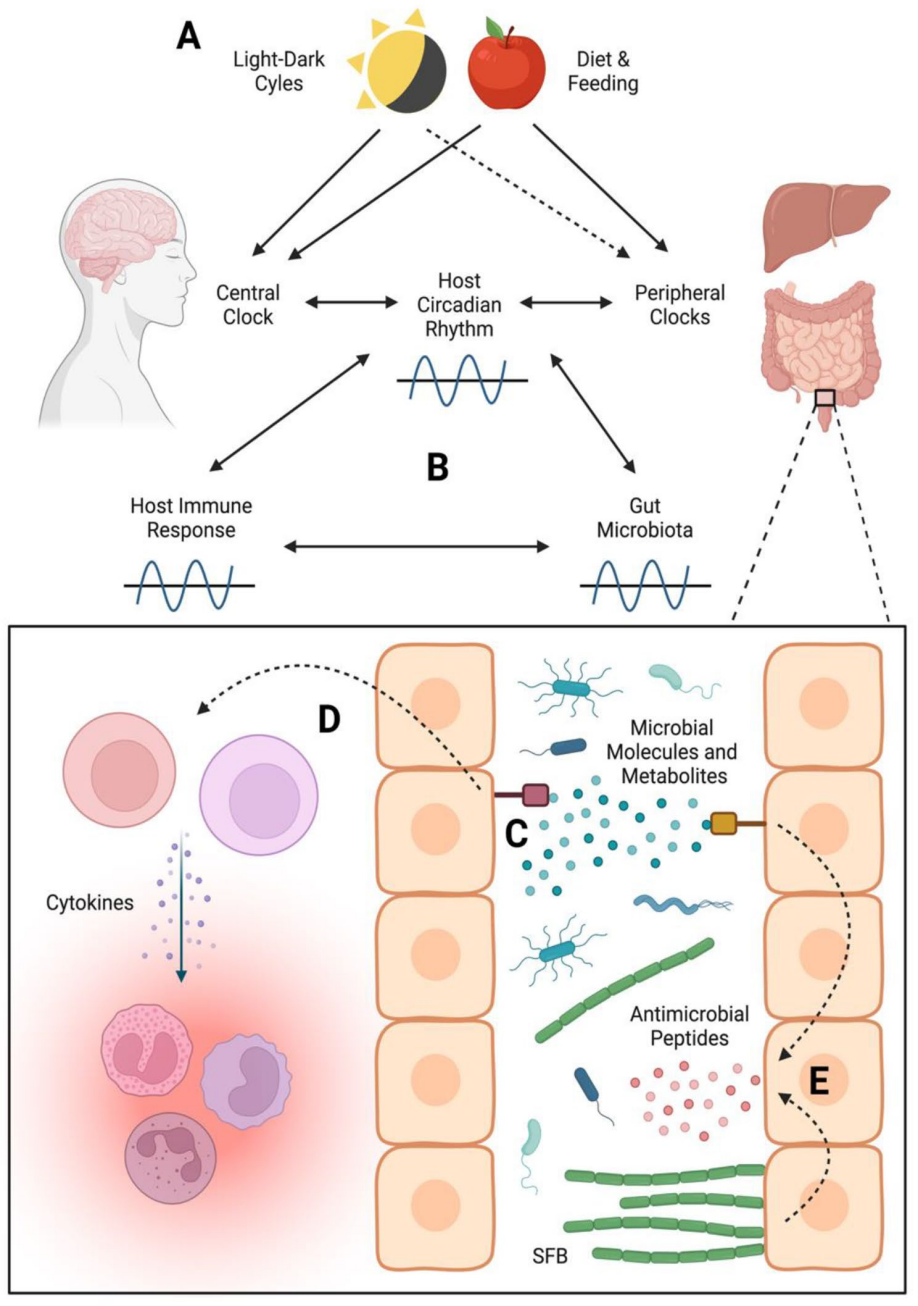
Interactions between host and gut microbiota diel cycles (A) Abiotic inputs including light-dark cycles and feeding habits entrain the host central clock (the circadian oscillator located in the SCN in the brain). Feeding directly impacts oscillations in gene expression in the peripheral clocks located in the gut and liver. Peripheral clocks are indirectly entrained to light through signals from the SCN. Together the central clock and peripheral clocks regulate the host circadian rhythm. (B) Host immune responses and gut microbiota display daily oscillations in function, magnitude, and activity. These oscillations impact and are influenced by the host circadian rhythm. (C) In the gut, molecules found on resident bacteria (e.g., flagellin and LPS) and microbial metabolites (e.g., BSHs, SCFAs, etc.) interact with host cell receptors. (D) These interactions can activate the host immune system and result in production of cytokines and ultimately inflammation in host tissues or (E) can trigger intestinal epithelial cells to produce antimicrobial peptides. SFB stimulate production of antimicrobial peptides by rhythmic interactions with host tissues, and regularly attach to gut epithelial cells in the morning. Resident microbiota and host cells influence each other in a complex and bidirectional manner (C-E). The behaviors of resident microbiota impact the host immune response and stimulate production of antimicrobial peptides, which ultimately impacts the physiology of the host as well as the species composition and metabolism of the gut microbiota. Created with BioRender.com.

The diverse microorganisms that inhabit or pass through the gastrointestinal (GI) tract of a vertebrate animal are known to change over the course of the life of the animal [112–115]. At any given moment, the composition of the gut community may shift as it is continually under the influence of various abiotic factors [116–118]. Light-dark cycles and host feeding schedule both impact host physiology and the gut microbial community, but the mechanisms by which light and feeding impact host circadian cycles differ (Fig. 3A). The mammalian circadian clock system is primarily controlled by a master timekeeper located in the hypothalamic suprachiasmatic nuclei (SCN). Light signals that enter the eye are relayed to the SCN via the retinohypothalamic tract. The SCN transmits timekeeping information to peripheral clocks in other regions of the brain or organs [119]. Feeding can influence peripheral clocks by means independent of the SCN, such as by altering the activity of metabolic-state dependent enzymes that impact the activity of key clock regulators [1]. Because light impacts the central clock system most directly, alterations to light-dark cycles are particularly important for control of circadian rhythms. While light appears to be the primary input for the SCN, other factors, such as melatonin, diet, physical movement, and even gut microbial metabolites may impact SCN hormonal and neuronal activities [120–124].

There is growing evidence that gut microbial community composition is altered by changes in light-dark cycles. Mice exposed to constant light show a decrease in gut microbiota alpha diversity and changes in the relative abundance of several taxa, including an increase in *Ruminococcus torques* abundance and a decrease in abundance of *Lactobacillus johnsonii*, aberrations that are correlated with decreased gut barrier integrity [125]. Rodents in constant darkness also exhibit a drop in gut microbial community alpha diversity and a loss of diel oscillations normally seen in gut microbiota composition [126, 127]. In addition, constant darkness results in an increase in the relative abundance of the class Clostridia in the small intestine of mice [126]. Constant light or darkness was also associated with an increase in the relative abundance of other genera including *Blautia*, *Prevotella*, *Lactobacillus* and *Bacteroides* and a decrease in *Parabacteroides* [127]. The implications of such changes are yet to be fully understood. As is often the case with circadian-rhythm-related research, in these studies, mice were allowed to feed *ad libitum*. It is possible that changes in the gut microbiota composition during alterations to light-dark cycles may be in part a result of feeding behavior changes rather than light-dependent molecular mechanisms.

From the perspective of the host, the deleterious impact of circadian disruption caused by alteration in light-dark cycles is well evidenced. Mice exposed to continuous light suffer a variety of issues including reduced skeletal muscle function, bone deterioration, and increased cytokine secretion resulting in a transient pro-inflammatory state [128]. These changes impact host response to bacterial invaders; mice in constant darkness show an increased risk of endotoxic shock triggered by bacterial lipopolysaccharide (LPS), a component of gram-negative bacterial cell envelope [129]. Disruptions to the normal sleep cycle have been shown to suppress chemokines that regulate inflammation and maintain the gut mucosal immunity barrier in mice, suggestive of a potential avenue for interaction between light-dark cycle disruptions and the gut microbiota [130]. In humans, the link between shifts in light-dark cycle and the prevalence of metabolic syndrome and obesity is well documented [131, 132]. In one study, germ-free mice that received a fecal transfer from time-shifted mice or jet-lagged humans exhibited increased weight gain and glucose intolerance, suggesting that the gut microbiota play a role in this pathology [133].

Circadian disruptions and shiftwork have also been shown to impact the human immune response, possibly by altering immune cell migration or cytokine secretions from monocytes and macrophages [134]. Light is particularly important for the regulation of group 3 innate lymphoid cells (ILC3s)—immune cells that produce cytokines important for stimulating the immune response—because reversal of light-dark cycles are known to alter the oscillatory expression of circadian clock genes in these cells [135]. In knockout mice where a key circadian feedback loop transcription factor is absent from ILC3s, changes in the expression of key clock genes, including *Clock*, *Cry1*, *Per3*, *Nr1d1* and *Nr1d2*, and increased apoptosis is observed, demonstrating the importance of the circadian clock system in regulating proper immune function [135–137]. In addition, rhythmic expression of core clock genes in ILC3s is absent in mice that have undergone surgical ablation of the SCN, supporting the assertion that light entrainment plays an important role [135]. Function of ILC3s is not solely controlled by light; it is also impacted by microbial signaling as suggested by the reduction in expression of an ILC3 in antibiotic-treated mice [138]. These findings suggest that the interaction between the host immune system and the gut microbiota may drive the changes to the gut microbial community associated with alterations in light-dark cycles. Further, changes in host immune response caused by a variety of stimuli may ultimately alter the gut microbiota composition and its physiological impacts on the host.

As evidenced by these studies, attempting to determine why gut microbial communities oscillate in response to light-dark cycles is technically challenging. The influence of host sleep-wake cycles, feeding schedule, and other factors likely impact diel oscillations of gut microbes complicating the study of these patterns. In addition, the host immune response displays cyclical changes driven by the impact of light on host circadian rhythms which may ultimately influence the gut microbiota, suggesting one possible mechanism for light-driven changes to the composition of microbial gut communities.

## Diet and feeding influence gut microbial community composition

The gut microbiota and host diet are so closely intertwined that it has been suggested that manipulation of diet alone could be an effective technique for engineering the gut microbiome [139]. Diet early in the life of an animal has a lasting influence on gut microbiota in adulthood and ultimately the health of the organism [140, 141]. While many studies have observed diel changes in gut microbiota composition in individual animals [23, 24, 126, 142], the overwhelming influence of diet on gut communities often confounds conclusions about the degree to which these changes may be controlled by host or potential bacterial circadian rhythms.

The microbial community composition within the rumen of dairy cows has been intensely studied with the promise of improving feed use efficiency and animal productivity through understanding, and perhaps manipulating, community composition and function [143]. Remarkably, samples taken from different cows at the same time of day (shortly after feeding) display less variation in the rumen community composition compared to samples taken from an individual cow throughout the day. This observation indicates the strong influence of feeding on the composition of the rumen microbiota [142]. Among diverse organisms, daily changes to the gut community composition have often been linked to feeding patterns, the processing of food as it passes through the gut, and diet. Intake of food can alter the gut community by introducing new organisms and by altering the abundance of existing community members [144, 145]. Factors including frequency of meals and overnight-fast duration have been shown to impact the human gut microbiome composition [146].

The type of diet and quantity of food eaten also appear to influence gut microbiota composition [147]. In humans, underfeeding results in an increase in the relative abundance of organisms in the phylum Verrucomicrobiota (synonym Verrucomicrobia) and reduces the capacity of the microbiota to extract nutrients [148]. Atypical diets—such as high fat diets—have been shown to reduce the alpha diversity of the gut microbiota and to result in fewer microbial species displaying diel oscillation patterns of relative abundance [23, 24, 149]. However, it has been observed that the consequences of abnormal diet types may be partially counteracted when feeding times are restricted to times of day when the host is active [24, 150]. This demonstrates the potential importance of time of feeding and feeding frequency to gut microbiota composition. In fact, mice fed a high fat diet and restricted to feeding at night, during their active period, show a significant increase in the number of species of gut bacteria displaying diel patterns of relative abundance compared to mice that are fed a high fat diet *ad libitum* [24]. In humans, fasting during the day and eating only at night results in significantly different gut community composition than unrestricted eating [151]. However, it is relevant to consider that host feeding times are not independent of the host circadian rhythm, thus examination of knockout mice where the circadian clock is attenuated is imperative to understanding this relationship. In knockout mice where several key host circadian feedback loop transcription factors are absent, mice permitted to feed throughout the day were more prone to obesity compared to those restricted to feeding at night, suggesting the host circadian clock helps to maintain diel feeding rhythms [150]. Similarly, when zebrafish are held in constant darkness, timed feeding can entrain expression of circadian clock genes and several cell cycle genes [152].

Regardless of diet type or time of feeding, the alpha diversity of gut microbial communities appears to be transiently altered post-feeding, with an initial increase in alpha diversity followed by a steady decrease. These patterns have been observed in a variety of systems including dairy cows, mice, and clownfish [24, 142, 153]. In addition to community diversity, feeding has also been shown to alter the abundance of specific bacterial groups. For instance, high Bacteroidota abundance is strongly correlated with fasting, a phenomenon likely due to their ability to metabolize host-derived polysaccharides [154]. In both mice and toads, the abundance of Bacteroidota species in the gut has been shown to rise during periods of fasting and fall after feeding [24, 155]. Similarly, in a study of dairy cows, from morning (fasting) to evening (several hours post-feeding) the relative abundance of Bacteroidota species in the rumen drastically decreased from 43% to 13% [142]. In clownfish, some groups of gut bacteria such as *Photobacterium* also undergo drastic shifts in abundance while other taxa such as *Clostridium* are more stable [153]. These findings highlight that some gut-associated bacterial species appear to be more prone to diet-induced shifts in abundance than others.

It is important to note that studies showing correlations between community composition and feeding are not free of confounding variables. Some experiments involve consistently feeding the animals at the same time each day and may lack controls for variables such as light-dark cycles or the host circadian rhythm. However, post-feeding shifts in the species composition of the gut microbiota are well documented across diverse organisms [24, 142, 153, 155]. Thus, such oscillations in community composition are likely driven, at least in part, by the influx of nutrients from feeding. Further, the clear connection between feeding and oscillations in the abundance of species in the gut microbiota highlights the need for feeding-independent methods of studying diel changes to the gut microbiota. Such methods may be required to fully disentangle the impact of physiological changes occurring due to host circadian rhythms from the changes brought about by host feeding behavior. Finally, there is evidence that community composition may be more substantially altered in cases where multiple disruptions occur simultaneously, such as when changes in host feeding rhythms are paired with antibiotic treatment or high-fat diet [156], further complicating the study of these complex and interconnected factors.

## Diet and feeding influence gut microbial metabolism and host cellular processes

In addition to altering the composition of the gut microbiota, diet and feeding significantly impact the metabolic activity of the gut microbiota and a variety of host processes [157]. Host fatty acid and lipid metabolism and host immune function are among the many processes altered by diet [158, 159].

For example, a ketogenic diet—which is high in fat and protein and low in carbohydrates—results in an increase in circulating ketone bodies in the host. This increase in ketone bodies has been shown to significantly alter the gut microbiota composition in mice, in particular with a reduction in *Bifidobacterium* abundance [160]. The decrease in *Bifidobacterium* is correlated with reduced levels of Th17 T-helper cells in the murine intestine, suggesting another potential link between the gut microbiota and host immunity, and highlighting the role of diet-induced changes to the gut microbiota in regulating host physiology [160]. In fact, a ketogenic diet has been shown to impact expression of host circadian clock genes and to activate the peroxisome proliferator-activated receptor alpha (PPARα) pathway which regulates host lipid metabolism in hepatocytes and intestinal cells in mice [161]. In the liver of ketogenic-diet-fed mice, the clock transcription factor BMAL1 shows changes in its recruitment of chromatin, a molecular mechanism for the regulation of gene expression, throughout the day [161]. Further, the amplitude of oscillations in gene expression of clock-controlled genes increased to a greater extent in the liver than in the gut of ketogenic-diet-fed mice. In addition, diel oscillations of PPARα protein expression occur in both the liver and gut, but the peak in PPARα expression in the liver is aligned with a trough in the gut [161]. This disconnect in the oscillation of protein and gene expression in the liver and gut highlights that peripheral clocks in different tissues may respond differently to changes in diet [161]. The role and potential mechanisms of the gut microbiota—and its changes resulting from diet—in regulating the gut peripheral clock has yet to be fully uncovered. However, there is growing evidence that such a link exists and is mediated by bacterial metabolites.

Short-chain fatty acids (SCFAs) are microbial fermentation products that result from microbial catabolism of host dietary carbohydrates. SCFAs provide energy for the host and can serve as signal molecules that alter host gene expression [162]. Shifts in the composition of SCFAs in the gut can be indicative of changes in metabolic activity or community composition of the gut microbiota [163]. In dairy cows, the start of carbohydrate fermentation following a meal results in an increase in the concentrations of SCFAs in the rumen and an increase in the number of glycolysis, pyruvate metabolism, and butyrate metabolism genes encoded by the rumen microbial community [142]. Some studies have examined changes to SCFA products and corresponding changes in both microbiota and host gene expression based on diet type. In rats, the concentration of the SCFA butyrate was significantly lower in individuals fed a high-protein diet and they exhibited down-regulation of genes involved in innate immunity and oxidative phosphorylation [164]. For humans, animal-based diets result in significantly lower concentrations of SCFAs associated with carbohydrate fermentation and higher levels of SCFAs associated with amino acid fermentation, along with increases in expression of genes related to vitamin biosynthesis and the production of β-lactamases by the gut microbiota compared to plant-based diets [163].

A recent study found that a high-fat diet is the primary driver of oscillations in abundance of several gut microbiota members which ultimately impacts antimicrobial peptide production by gut epithelial cells [149]. A high-fat diet was found to diminish oscillations in gut microbiota composition and promoted higher abundance of Clostridiaceae, Peptostreptococcaceae, and some *Lactobacillus* species in the gut of mice. These changes in community composition were paired with reduced cyclical expression of the antimicrobial peptide Reg3γ in the gut [149]. Reg3γ expression was found to be regulated by small molecules produced by several gut bacteria, including *Lactobacillus rhamnosus* GG which induces expression and *Peptoanaerobacter stomatis* (Family Peptostreptococcaceae) which suppresses expression [149]. Mechanisms such as the regulation of host gene expression by bacterial metabolites provide some insight into how oscillations in the composition and metabolic activities of gut microbiota and host behavior and physiology may be intertwined: host diet impacts the composition of the gut microbiota, which impacts production of antimicrobial peptides by host cells (Fig. 3E), in turn potentially further altering or maintaining the composition of the gut microbiota.

As is also supported by many of these studies, the host immune response and metabolic processes can be altered by diet, food intake, and gut microbiota [165, 166]. Both adaptive and innate immune functions have been shown to oscillate on a diel basis [167]. The impact of diet on host metabolic processes and immune response paired with the role of host circadian clock in regulating the immune response, raises another relevant factor in understanding the relationship between the host and the gut microbiota. Because host cells display diel changes related to immune function and metabolism, it is challenging to disentangle host circadian controlled systems from those controlled by the oscillations of microbial gut symbionts (Fig. 3B).

## Gut bacteria respond to rhythmic cues from host behaviors and metabolic cycles

For mammals, a vast array of host behaviors ranging from sleep-wake cycles to eating cooked food can influence the gut microbiome [168, 169]. Therefore, it is not surprising that the host circadian rhythm impacts the gut microbiota. Both the central circadian clock, housed in the SCN, and peripheral clocks play a role in regulating the gut microbiota [167]. In fact, the tissue of the GI tract possesses a peripheral clock system of its own [170] providing an interface for interaction between the gut microbiota and the host circadian clock. Circadian rhythms regulate many GI functions including colonic motility, gastric acid production, and gut barrier function [170]. Coordination between the intestinal circadian clock and gut microbiota is important for homeostasis of the intestinal epithelium and host health [171].

The interaction between the host circadian clock and gut microbiota influences host metabolism [172, 173]. In mice, circadian disruption either caused by alterations to the light-dark cycle or deletion of important clock genes in the animal, resulted in both abnormal diel fluctuations of the gut microbiota and dysbiosis. Microorganisms that typically display rhythmic diel fluctuations in abundance in wild-type mice instead showed random abundance fluctuations in clock gene knockout mice [133]. Functions including vitamin metabolism, nucleotide metabolism, DNA repair, cell wall synthesis, and motility that exhibited rhythmic oscillations in abundance in the metagenomes of the gut microbiota of wild-type mice did not show rhythmic oscillation in clock gene knockout mice, indicating the importance of host circadian rhythms in maintaining both diel gut microbiota compositional and metabolic patterns [133]. There is evidence that the prevalence and gene expression of some gut bacteria also oscillates on a diel basis in humans, suggesting that the host circadian rhythm may alter the gut microbiome and, in turn, affect host metabolism and weight [133, 174].

One factor that facilitates this intricate relationship between the host and its gut microbiota is that gut bacteria respond to diel cycles of secreted host hormones in the gut. The hormone melatonin has been strongly linked to regulating the sleep-wake cycle in humans [175]. Melatonin is commonly found in many foods and in the gut environment. Host-derived melatonin is secreted into the lumen of the gut after being produced in the pineal gland [175]. Certain gut bacteria have been shown to react to melatonin. In *Klebsiella aerogenes*, melatonin has been shown to increase swarming motility and to synchronize the expression of the motor protein gene *motA* to a roughly daily cycle [56, 57]. Although the authors of these in vitro studies suggest endogenous bacterial circadian rhythms, in vivo it is difficult to separate the impact of the host circadian-controlled production of melatonin from reactions by the gut microbial populations. Further, the molecular mechanisms for such diel oscillations in heterotrophic bacteria within the gut have yet to be uncovered.

Glucocorticoids, a group of steroid hormones which can be produced by intestinal epithelial cells, also show a diel secretion pattern [176]. Imbalance of glucocorticoids has been observed to alter host expression of circadian clock-related genes and to change the composition of the gut microbiota, reducing alpha diversity and decreasing the relative abundance of the phyla Bacillota (synonym Firmicutes), Bacteroidota, Pseudomonadota, and Actinomycetota in rats [177]. Glucocorticoids are commonly used to treat a variety of inflammatory or autoimmune diseases including inflammatory bowel disease (IBD), multiple sclerosis, rheumatoid arthritis, and asthma [176]. Strikingly, these conditions have been linked to dysbiosis of the gut microbiome [178]. These findings indicate a relationship between host diel hormone secretion and the behavior and composition of the gut microbial community, suggesting a mechanism by which the host influences and regulates resident gut microbiota.

The apparent synchrony between the microbiota and host may be advantageous to both parties. However, pathogens able to synchronize their metabolism, growth cycle, or other behaviors with host rhythms could use these signals to optimize their infectivity. Knockout mice lacking a core circadian clock gene were less susceptible to infection by the bacterium *Streptococcus pneumoniae*—the leading cause of community-aquired pneumonia—than mice with a fully intact circadian clock system [179]. While this study also found that knockout mice displayed increased macrophage motility and phagocytic function, likely explaining this observation, it did not attempt to quantify metabolic or functional changes in the pathogen [179]. Similarly, human cells with a disrupted circadian clock system, infected with flaviviruses—including the hepatitis C virus, dengue, and Zika—showed a marked decrease in viral replication compared to wildtype cells [180]. However, the opposite effect has been observed for other viral challenges. Knockout mice infected with Sendai virus and influenza A show exacerbated acute viral bronchiolitis and high viral load [181]. Similar dynamics may also be important in gut systems, and there is some evidence that time of infection impacts disease outcome. Mice infected with *Salmonella enterica* serovar Typhimurium in the morning harbor a larger pathogen population and develop a greater proinflammatory response compared with mice infected at other times of day [182]. While there is some evidence of the importance of circadian rhythms in influencing disease outcomes, further study is necessary to determine whether pathogens can synchronize with host circadian rhythmns and whether this ability might provide an evolutionary advantage to the pathogen.

## Gut microbiota influence host circadian rhythms

Although the gut microbiota is heavily influenced by host physiology and behavior, gut microbes also influence the health of their host, from weight maintenance to cognitive development [183]. Correlations between gut microbiota composition, host circadian rhythm, and a variety of psychiatric and physical health conditions, suggest that the interaction between the gut microbiota and host circadian rhythms may be important for human health [178, 184–186].

Several studies have demonstrated a link between the gut microbiota, the cyclical expression of host genes, and oscillations in the abundance of gut metabolites. The role of the gut microbiota in influencing these transcriptional and metabolic patterns can be studied by using antibiotic-treated and germ-free mice to examine the impact of the depletion or absence of gut bacteria. In antibiotic-treated and germ-free mice, the rhythmicity of various intestinal metabolites—including amino acids and polyamines—ceases, suggesting that changes in the abundance of some gut metabolites are mediated by the gut microbiota [187]. Antibiotic-treated and germ-free mice also show substantial alterations to oscillations in the expression of host transcripts in both the intestine and liver [187, 188]. Although the expression of host core clock genes in these tissues do not appear to be significantly altered in antibiotic-treated or germ-free mice, the depletion or absence of the gut microbiota results in a loss of rhythmicity of expression for various host genes mainly involved in nucleotide metabolism and cell-cycle pathways [187]. This loss of detectable rhythmicity in the expression of various genes in the liver suggests that the gut microbiota may play an important role in regulating rhythmic gene expression in peripheral tissues in addition to the gut [187]. Although many genes lose rhythmicity in the absence of the gut microbiota, strikingly, key metabolic pathways that typically display transcriptional oscillations in the microbiota gain rhythmicity in host colonic tissue when the gut microbiota are depleted [187]. These findings suggest that in the absence of the microbiota, the host may compensate by carrying out processes that are commonly performed by the microbiota [187].

As suggested above, one mechanism for microbial influence on host rhythms is through the control of gut metabolites, including the production of microbial metabolites (Fig. 3C). Microbial fermentation products, including the SCFAs acetate, butyrate, and propionate, and the organic acid lactate, have been linked with altered host circadian rhythms. Studies have shown that oral administration of acetate, butyrate, propionate, and lactate causes changes to the expression of clock-related genes in both mice and rats [164, 189]. Similar results have been observed in other studies where changes in the concentration of the SCFA butyrate in the gut are correlated with changes in the rhythmicity of expression of host circadian clock genes [23, 114, 164]. Bile salt hydrolase (BSH) produced by gut bacteria is important for the conversion of bile salts into secondary bile acids which have been shown to alter the expression of several genes involved in regulating host circadian rhythms [190]. Trimethylamine, which is produced by the gut microbiota from dietary choline, is another potential regulator of the host circadian clock. Mice treated with trimethylamine lyase to inhibit the activity of trimethylamine showed alterations to the expression of various clock genes [191]. In addition, bacteria are known to produce a variety of neurotransmitters including serotonin, acetylcholine, and gamma-aminobutyric acid (GABA) [192]. Many of these neurotransmitters may play a role in regulating host circadian rhythms [193] suggesting another possible mechanism by which microbial metabolites may influence the host circadian rhythm.

Despite the correlations observed between specific microbial metabolites and host circadian rhythms, the molecular basis for the relationship between the gut microbiota and host circadian clock is poorly understood. Recently, one potential mechanism for this interaction has emerged. Histone deacetylase 3 (HDAC3) plays a critical role in transcriptional regulation of the mammalian circadian rhythm and glucose metabolism [194]. Gut bacteria appear to modulate rhythmic expression of *Hdac3* in epithelial cells. Germ-free mice do not exhibit the normal oscillations in histone acetylation observed in conventional mice. These oscillations correlate with enrichment of host genes involved in metabolic processes including nutrient transport and lipid metabolism, suggesting that the microbiota play a role in regulating these processes [195].

Interaction between the host immune system and gut bacteria is also relevant to the interaction between gut microbiota and the host circadian clock [165]. Recent work has indicated that this relationship is facilitated by microbial signals relayed to the host via receptors on epithelial cells (Fig. 3C). The interaction between bacterial metabolites or cell components—such as LPS and flagellin—and Toll-like receptors (TLRs) in intestinal epithelial cells is important for intestinal homeostasis [196]. In intestinal epithelial cells, bacterial signals transduced by TLRs influence expression of circadian clock genes [171]. The diel oscillation of the epithelial cell circadian transcription factor *Nfil3*, which regulates host lipid uptake, is modulated by bacteria and is expressed at a notably lower level in germ-free mice, indicating that gut microbiota are necessary to maintain proper circadian function [197]. The transcription factor signal transducer and activator of transcription 3 (STAT3) pathway provides a mechanism for the interaction of the gut microbiota with the epithelial circadian clock via TLRs. Gut bacteria interact with TLRs on dendritic cells resulting in cytokine production (Fig. 3D). Cytokines activate the ILC3s which produce another cytokine—interleukin 22 (IL-22)—that activates STAT3 in epithelial cells ultimately fine-tuning the amplitude of expression of *Nfil3* [197]. Because flagellin and LPS interact with TLRs, gram-negative and motile bacteria can activate the STAT3 pathway. Indeed, treatment of germ-free mice with flagellin and LPS alters expression of *Nfil3* and other clock genes [197].

In mice, one group of bacteria in particular—the segmented filamentous bacteria—have been shown to directly influence the host circadian clock via the STAT3 pathway. Segmented filamentous bacteria (SFB) attach to the intestinal epithelia at dawn controlling diel rhythms of STAT3 expression which in turn controls oscillation in antimicrobial peptide expression by the epithelium (Fig. 3E), providing a possible mechanism for ultimately altering the composition and function of the gut microbiota throughout the day [198]. However, the attachment rhythms of SFB are not feeding-independent; day-fed mice display a 12-hour phase shift in the SFB attachment peak compared to night-fed mice [198]. Thus, feeding time significantly drives SFB attachment timing and the resulting changes in *Stat3* expression, highlighting the role of host behavior in shaping the rhythms of gut microbiota. Regardless, SFB attachment behavior may play a significant role in host health. The authors suggest that SFB may impact host susceptibility to foodborne pathogens, observing that time-dependent susceptibility to *S.* Typhimurium was eliminated when SFB abundance was reduced by treatment with streptomycin [198].

Together these recent mechanistic studies suggest that the gut microbiota may play a more active role than previously thought in modulating host circadian rhythms by a variety of molecular mechanisms with the host immune system being a particularly important interface. Despite these recent breakthroughs, a great deal of further study is necessary to fully elucidate the complex mechanisms for interactions between gut bacteria and the host circadian clock.

## Conclusions

Like all life on Earth, microbes are impacted by the diel cycles that arise from the rotation of our planet about its axis. Although the only characterized circadian clock mechanism in bacteria to date is the Kai system in cyanobacteria, diverse bacteria undergo oscillations in gene expression on a diel basis. For example, heterotrophic bacteria in aquatic systems are impacted by daily light-dark cycles and shifts in available nutrients that correspond with the photosynthetic activity of nearby microbial phototrophs [199, 200]. These metabolic handoffs are relevant to the ecology of microbial communities and bacterial populations in a diverse range of environments, including in host-associated systems. Further, it is possible that the diel cycles in metabolism displayed by one member of a microbial community may modulate the cycles of neighboring microorganisms. For example, if some species of gut symbionts possess their own endogenous rhythms, these species may play a role in controlling diel oscillations in the abundance of various other members of the gut bacterial community.

A comprehensive understanding of the relationship between animals and their resident gut microbiota has yet to be achieved. Bacteria appear to play a role in modulating host diel cycles through the metabolites they produce that impact the expression of host genes involved in the transcriptional-translational feedback loops that control the host circadian rhythm. Conversely, bacteria also respond to host hormone cycles as well as environmental inputs such as nutrients from host feeding. This complex relationship will require further research to fully explain how gut bacteria and host physiology each play a role in modulating the circadian rhythms that have been observed among life on Earth.

In humans, limitations on the ability to collect fecal or other gut samples from one individual at sufficient timepoints throughout the day presents a problem, making observation of diel patterns in the human gut microbiota difficult. Regardless, there is evidence that human behaviors can shift the gut microbiota on a daily basis [201]. In addition, several studies have attempted to bypass this issue by collecting samples at various timepoints throughout the day among small cohorts or by comparing groups of time-stamped samples from different individuals [133, 174]. Despite the technical challenges, based on the diel changes in the gut microbiome seen in animal models, natural systems, and humans described in this review, it is evident that diel shifts influence organism health.

As outlined throughout this review, there is a great deal of evidence that gut bacteria respond to cyclical changes in the gut environment caused by host behaviors such as feeding and that changes in the host circadian rhythm brought on by changes in sleep pattern or light-dark cycles impact the composition of the gut microbiota. Conversely, changes in gut microbiota composition can influence host gene expression and impact the circadian rhythm of the host. Specific molecular mechanisms by which several gut bacterial species interact with and influence their hosts have been recently uncovered. While it is clear that the gut microbiota plays an active role in modulating host circadian rhythms, changes in the abundance of influential species can often be explained by host behaviors such as feeding schedule and diet.

Nevertheless, the molecular mechanisms by which heterotrophic bacteria may regulate their own circadian rhythms requires further study. If and how novel endogenous clock systems function across the diverse phyla of the bacterial domain of life remains unclear. While it may be evolutionary advantageous for bacteria to possess a circadian clock to both respond to predictable daily environmental changes and for host-associated bacteria to anticipate the cyclical behaviors of their host, further evidence is needed to confirm or reject this hypothesis.

## Data Availability

Data sharing is not applicable to this article as no datasets were generated or analyzed.

## Abbreviations

SCFAs: Short-chain fatty acids
MIF: Macrophage migration inhibitory factor
PPARα: peroxisome proliferator-activated receptor alpha
ILC3s: group 3 innate lymphoid cells
SCN: hypothalamic suprachiasmatic nuclei
HPA: hypothalamic-pituitary-adrenal
IBD: inflammatory bowel disease
GI: gastrointestinal
BSH: bile salt hydrolase
GABA: gamma-aminobutyric acid
PPD: 1-phenyl-1,2-propanedione
STAT3: signal transducer and activator of transcription 3
LPS: lipopolysaccharide
TLRs: Toll-like receptors
HDAC3: histone deacetylase 3
IL-22: interleukin 22
SFB: segmented filamentous bacteria

## Glossary

*Circadian rhythm*: A self-sustained, endogenous biological cycle that occur on an approximately 24-hour period and is entrained to environmental stimuli but maintains its period even with shifts in temperature.
*Circadian clock*: The set of molecular mechanisms by which a circadian rhythm is maintained.
*Entrainment*: The process by which external rhythmic cues coordinate and reinforce the cycle of an internal circadian clock.
*Diel cycle*: A cycle occurring over a 24-hour period which is not necessarily endogenous or self-sustained. The term diurnal cycle is sometimes used in the literature to describe daily cyclical changes in abundance or metabolism among the gut microbiota. Because diurnal can also be used to refer to organisms that are active during the day, for clarity we use diel throughout this review.
*Heterotroph*: An organism that derives energy from organic matter.
*Phototroph*: An organism that can harness energy from light to drive metabolic functions.
*Bacterioplankton*: The bacterial component of the plankton.
*Dysbiosis*: An imbalance in composition or function of the gut microbial community that is often associated with disease.
